# Strategies for involving patients and the public in scaling-up initiatives in health and social services: protocol for a scoping review and Delphi survey

**DOI:** 10.1186/s13643-021-01597-6

**Published:** 2021-02-11

**Authors:** Ali Ben Charif, Karine V. Plourde, Sabrina Guay-Bélanger, Hervé Tchala Vignon Zomahoun, Amédé Gogovor, Sharon Straus, Ron Beleno, Kathy Kastner, Robert K. D. McLean, Andrew J. Milat, Luke Wolfenden, Jean-Sébastien Paquette, Friedemann Geiger, France Légaré, The RePOS Network, Martin Beaumont, Ron Beleno, Ali Ben Charif, Arlene Bierman, Johanne Blais, Carol Fancott, Friedemann Geiger, Amédé Gogovor, Sabrina Guay-Bélanger, Kathy Kastner, France Légaré, Robert McLean, Andrew J. Milat, Jean-Sébastien Paquette, Karine V. Plourde, Francois Rivest, Sharon Straus, Guy Thibodeau, Luke Wolfenden, Hervé Tchala Vignon Zomahoun

**Affiliations:** 1 VITAM–Centre de recherche en santé durable, Université Laval, Pavillon Landry-Poulin, 2525, Chemin de la Canardière, Quebec City, QC G1J 0A4 Canada; 2 Tier 1 Canada Research Chair in Shared Decision Making and Knowledge Translation, Université Laval, Quebec City, QC Canada; 3 Department of Family Medicine and Emergency Medicine, Université Laval, Quebec City, QC Canada; 4 Health and Social Services Systems, Knowledge Translation and Implementation component of the Quebec SPOR-SUPPORT Unit, Université Laval, Quebec City, QC Canada; 5 Faculty of Medicine, School of Physical and Occupational Therapy, Epidemiology, Biostatistics, and Occupational Health, McGill University, Montreal, QC Canada; 6 LiKaShing Knowledge Institute, St. Michael’s Hospital, Toronto, ON Canada; 7 St. Michael’s Hospital, Toronto, ON Canada; 8 Department of Medicine, University of Toronto, Toronto, ON Canada; 9 Age-Well NCE, Toronto Rehabilitation Institute, Toronto, ON Canada; 10 Best Endings, Toronto, ON Canada; 11 International Development Research Centre, Ottawa, ON Canada; 12 Faculty of Medicine and Health Sciences, Stellenbosch University, Tygerberg, South Africa; 13 School of Public Health, University of Sydney, Sydney, NSW Australia; 14 Centre for Epidemiology and Evidence, NSW Ministry of Health, Sydney, NSW Australia; 15 School of Medicine and Public Health, University of Newcastle, Callaghan, NSW Australia; 16 Hunter Medical Research Institute, New Lambton Heights, NSW Australia; 17 Hunter New England Population Health, Wallsend, NSW Australia; 18 Laboratoire ARIMED, Groupe de médecine de famille universitaire (GMF-U) de Saint-Charles-Borromée, Saint-Charles-Borromée, QC Canada; 19 SHARE TO CARE Project, University Medical Center Schleswig-Holstein (UKSH), Kiel, Germany; 20 Department of Pediatrics, University Medical Center Schleswig-Holstein (UKSH), Kiel, Germany; 21 Institute for Medical Psychology and Medical Sociology, University Medical Center Schleswig-Holstein (UKSH), Kiel, Germany; 22 Department of Psychology, MSH Medical School Hamburg, Hamburg, Germany; 23 Population Health and Practice-Changing Research Group, CHU de Québec Research Centre, Quebec City, QC Canada; 24 Diabetes Action Canada, a SPOR Network in Diabetes and its Related Complications, Université Laval, Quebec City, QC Canada

**Keywords:** Patient and public involvement, Scaling up, Spread, Scaling science, Health and social services, Implementation science, Knowledge translation, Strategy

## Abstract

**Background:**

The scale-up of evidence-based innovations is required to reduce waste and inequities in health and social services (HSS). However, it often tends to be a top-down process initiated by policy makers, and the values of the intended beneficiaries are forgotten. Involving multiple stakeholders including patients and the public in the scaling-up process is thus essential but highly complex. We propose to identify relevant strategies for meaningfully and equitably involving patients and the public in the science and practice of scaling up in HSS.

**Methods:**

We will adapt our overall method from the RAND/UCLA Appropriateness Method. Following this, we will perform a two-prong study design (knowledge synthesis and Delphi study) grounded in an integrated knowledge translation approach. This approach involves extensive participation of a network of stakeholders interested in patient and public involvement (PPI) in scaling up and a multidisciplinary steering committee. We will conduct a systematic scoping review following the methodology recommended in the Joanna Briggs Institute Reviewers Manual. We will use the following eligibility criteria: (1) participants—any stakeholder involved in creating or testing a strategy for PPI; (2) intervention—any PPI strategy proposed for scaling-up initiatives; (3) comparator—no restriction; (4) outcomes: any process or outcome metrics related to PPI; and (5) setting—HSS. We will search electronic databases (e.g., Medline, Web of Science, Sociological Abstract) from inception onwards, hand search relevant websites, screen the reference lists of included records, and consult experts in the field. Two reviewers will independently select and extract eligible studies. We will summarize data quantitatively and qualitatively and report results using the PRISMA extension for Scoping Reviews (PRISMA-ScR) checklist. We will conduct an online Delphi survey to achieve consensus on the relevant strategies for PPI in scaling-up initiatives in HSS. Participants will include stakeholders from low-, middle-, and high-income countries. We anticipate that three rounds will allow an acceptable degree of agreement on research priorities.

**Discussion:**

Our findings will advance understanding of how to meaningfully and equitably involve patients and the public in scaling-up initiatives for sustainable HSS.

**Systematic review registration:**

We registered this protocol with the Open Science Framework on August 19, 2020 (https://osf.io/zqpx7/).

**Supplementary Information:**

The online version contains supplementary material available at 10.1186/s13643-021-01597-6.

## Background

There is widespread enthusiasm for the use of ideas, practices, or products perceived as new (i.e., innovations) and for which an evidence base has been established (i.e., evidence-based innovations) in order to improve health and social services (HSS) [1, 2]. The impact of evidence-based innovations (EBIs) should be optimized to reduce waste and inequities in HSS and improve the health of populations [3, 4]. But evidence is limited on how to implement EBIs so that they benefit more patients or a broader spectrum of patients in healthcare systems [5]. For example, there is a shortage of evidence on strategies for expanding or “scaling up” EBIs [6]. In knowledge translation (KT) and implementation science (both hereafter referred to as KT), the differences between “scaling up,” “scaling out,” “scaling deep,” “scaling,” and “spread” are variously nuanced [5–7]. Here, we use the term “scaling up” or “scale up,” which is more familiar to lay knowledge users (e.g., patients and the public). The World Health Organization defines the process of “scaling up” as “deliberate efforts to increase the impact of successfully tested health innovations so as to benefit more people and to foster policy and program development on a lasting basis” [8]. Here, we understand scaling up to optimize equity, for example, as enlarging the scope of EBI not only numerically but also in other respects, e.g., increasing its range to a wider variety of socio-economic backgrounds [5]. While anticipating scaling up should begin at the earliest stages of research [5, 9], we summarized the main scaling-up steps as follows [1]: (1) scalability assessment, (2) development of a scaling-up strategy, (3) implementation and evaluation of the strategy, and (4) promoting the long-term sustained use of the successfully scaled-up EBI. The scalability assessment, the preliminary and essential step in scaling up an EBI, refers to assessment of the “ability of a health innovation shown to be efficacious on a small scale and/or under controlled conditions to be expanded under real world conditions to reach a greater proportion [or range] of the eligible population, while retaining effectiveness” [5, 10].

Just as the scaling up of EBIs remains an understudied phase of KT [11, 12], the science and practice of scaling up, in turn, has not yet fully integrated patient-oriented research principles [6, 7]. Our previous knowledge synthesis shows that barriers to scaling up EBIs include an absence of patient and public involvement (PPI) [6]. Scaling up often tends to be a top-down process initiated by policy makers and/or researchers [6, 7]. The values, preferences, and perspectives of the intended beneficiaries, i.e., the patients and public, are often forgotten. As patients and the public are at the core of healthcare systems that serve them, it is essential that they be part of the scaling up of such systems [13, 14], including preparatory, execution, and translational phases [15]. Not only should they be involved, but their involvement should be meaningful and equitable, i.e., it should be more than tokenistic, and their input should carry equal weight as that of policy makers and researchers. Strengthening patient and public influence on health decisions at every level is a necessary condition for a continuously learning health care system [16, 17]. However, policy makers and researchers who want to involve lay participants in this domain dominated by experts face a multiple of challenges, which need to be addressed.

In this research, we aim to address this knowledge gap in KT by building patient-oriented research capacity in the science and practice of scaling up in HSS, by ensuring that patients and the public are meaningfully and equitably involved. Therefore, our research question is: “How can patients and the public be meaningfully and equitably involved in scaling-up initiatives in HSS?” Our specific objectives are to (1) review existing literature on PPI in any scaling-up initiative in HSS; (2) achieve consensus on relevant strategies for involving patients and the public at each scaling-up step in HSS; and (3) build an international network of interest in the involvement of patients, the public, and other stakeholders in the science and practice of scaling up in HSS. This effort will contribute to an evidence-driven and people-centered science of scaling up.

## Methods

### Overarching methodological approach

We will adapt our overall method from the RAND/UCLA Appropriateness Method [18, 19]. Following this, our research will entail a two-prong study design: (1) a knowledge synthesis and (2) a Delphi study. We will use the Canadian Institutes of Health Research (CIHR) guidance to integrate considerations of sex and gender [20] into both the knowledge synthesis and the Delphi study. Our project is grounded in an integrated KT (iKT) approach and thus involves extensive participation of (1) a network of stakeholders interested in PPI in scaling up, the Research on Patient-Oriented Scaling-up (RePOS) Network, and (2) a multidisciplinary steering committee. The latter will include at least two patients or citizens, one policy- or decision-maker, one health care provider, one researcher, one scientific coordinator, and one trainee. We will use the CIHR Model of Knowledge Translation [21], the Montreal model [14], and the Public and Patient Engagement Evaluation Tool (PPEET) [22] to guide and assess our iKT approach. Finally, to ensure accurate and transparent reporting of this iKT approach, we will report evaluations using the Guidance for Reporting Involvement of Patients and the Public (GRIPP2) checklist [23] and the Sex and Gender Equity in Research (SAGER) guidelines [24].

### Knowledge synthesis

#### Study design

We will conduct a scoping review to identify strategies that have been proposed for involving patients and the public in the science or practice of scaling up innovations in HSS. Scoping reviews are defined as a type of knowledge synthesis that follows a systematic approach to map evidence on a topic and identify main concepts, theories, sources, and knowledge gaps [25]. We will follow the methodology recommended in the Joanna Briggs Institute (JBI) Reviewers’ Manual for scoping reviews [26, 27] and have registered the protocol in the Open Science Framework (OSF) on August 19, 2020 (registration identifier: https://osf.io/zqpx7/) [28]. We report the content for this scoping review protocol using the Preferred Reporting Items for Systematic reviews and Meta-Analyses Protocol (PRISMA-P) checklist (Additional file 1) [29].

#### Eligibility criteria

Following the “PICOS” (participants, intervention, comparator, outcome, and setting) framework [30], we will use the following inclusion criteria:

- ▪ Participants (P): Any type of stakeholder involved in creating or testing a strategy for PPI in the science or practice of scaling up. Stakeholder refers to “individual or group who is responsible for or affected by health- and healthcare-related decisions that can be informed by research evidence” [31, 32]. We will include the following nine types of stakeholders: (1) *patients and the public* (e.g., patients, their informal caregivers, their families, patient and consumer advocacy organizations, and local, national, or global citizens), (2) *providers* (e.g., individuals and organizations that provide care to patients and populations such as nurses, physicians, pharmacists, mental health counselors, and community-based workers), (3) *purchasers* (e.g., employers, governments, and other individuals or entities responsible for underwriting the cost of care), (4) *payers* (e.g., payers who pay or reimburse costs of health-related interventions such as insurers, individuals with deductibles, and others responsible for reimbursement for health-related\ interventions), (5) *policy makers* (e.g., policymaking entities such as governments and professional associations), (6) *product makers* (e.g., drug and device manufacturers), (7) *investigators* (e.g., researchers, postdoctoral fellows, and research coordinators), (8) *the press* (e.g., publishers and news media), and (9) *trainees* (e.g., university or college students) [31–34]. This includes organizations that provide guidance for promoting PPI in the science and practice of scaling up in HSS.
- ▪ Intervention (I): Any strategy proposed for involving patients and/or the public (alone) or multiple stakeholders (including patients and/or the public) in research or practical initiatives related to the steps of the scaling-up process in HSS. We will consider the four levels of PPI [13, 14]: (1) information (i.e., patient or the public receives information but has no role in contributing); (2) consultation (i.e., patients or the public provide their views, thoughts, feedback, opinions, or experiences but without a commitment to act on them; (3) collaboration (i.e., patients or the public are engaged to influence the scaling-up initiative, including commenting, advising, ranking, voting, prioritizing, and reaching consensus, but without direct control over decisions; and (4) coproduction (i.e., patients or the public are equal members of the research team and participate in all steps of the scaling-up initiative).
- ▪ Comparator (C): We will consider for inclusion both studies with comparison group and studies without.
- ▪ Outcomes (O): We will consider a wide range of metrics related to PPI. We will use the following taxonomy: process metrics and outcome metrics [35]. The process metrics form four domains: (1) direct process metrics (e.g., agenda setting and time allocation, roles in decision making, and control over the meeting minutes); (2) surrogate process metrics (e.g., formal power, organizational commitment to involvement, and equality of participation); (3) preconditions for involvement metrics (e.g., literacy of patient and public participants, resources provided, and presence or quality of training); and (4) aggregate process metrics (e.g., respect, transparency of the decision-making process, and level or ladder of participation). The outcome metrics form three domains: (1) internal outcomes as measured by impact on involved patient or public participants (e.g., knowledge, satisfaction, and trust), on services provided (e.g., efficiency and cost-effectiveness of services, number of complaints about services, and services quality and safety), and on organization or system (e.g., funding and resources availability, staff views on PPI, and presence of racism in system); (2) external outcomes as measured by influence on the broader public (e.g., awareness or knowledge of health issues, support of the organization or system, and PPI as part of social change outside the organization), and on population health (e.g., population health status and level of health inequalities); and (3) aggregate outcomes (e.g., overall cost-effectiveness of PPI).
- ▪ Setting (S): Any context related to HSS. The World Health Organization defines health as “a state of complete physical, mental and social well-being and not merely the absence of disease or infirmity” [36].

#### Literature search

We will perform a comprehensive search for both published peer-reviewed records and “grey literature” through searching electronic databases, hand searching relevant websites, screening the reference lists of included records, and consulting experts in the field. *First*, we will search Medline, EMBASE, Web of Science, CINAHL, PsycINFO, ERIC, the Cochrane Library, Sociological Abstract, and Academic Search Premier from their inception onwards. Our information specialist will perform the electronic search strategy in Medline. The search terms will reflect the concepts: (1) PPI, (2) scaling up, and (3) HSS. These terms will be adapted to the above-mentioned databases. There will be no restriction with regard to language, date of publication, or type of document. This strategy will be reviewed by our international authors, experts on scaling up, and stakeholder involvement, then by a second information specialist using the Peer Review of Electronic Search Strategies (PRESS) guideline [37]. A draft of the search strategy can be found in Additional file 2. *Second*, we will consult the “grey literature” involving general and targeted Internet searches. This approach is promoted by the Agency for Healthcare Research and Quality (AHRQ) as a way of reducing publication bias [38]. Grey literature consists of unpublished literature, including publicly available information produced by all levels of government, academic institutions, business, and industry, in print and electronic formats, which is not controlled by commercial publishers [39–41]. For the general search, we will enter various configurations of a search term list into Google. For the targeted search, we will examine websites of the national and international organizations recommended by our steering committee and by other international scaling-up experts. Additionally, we will use the approach recommended by the Canadian Agency for Drugs and Technologies in Health (CADTH) for systematic searches of grey literature [42]. *Finally*, we will search published bibliographies of related topics and citations in included articles and consult experts in the field to identify additional relevant studies.

#### Study selection

We will operationalize our inclusion criteria using questions based on our PICOS elements. Two reviewers will perform a calibration exercise to ensure the criteria capture relevant studies. They will also independently screen titles and abstracts of a random sample of 5% of studies identified with our literature search. We will discuss the results of this pilot and review the eligibility criteria. We will check inter-reviewer agreement between these reviewers using the kappa statistic. Then, two reviewers will independently screen all remaining titles and abstracts for relevance, procure full texts of relevant studies, and select eligible studies. We will resolve any disagreements through consensus between the two reviewers and, if needed, with a third party. We will use the Covidence software for the selection of titles and abstracts [43]. Eligible studies will be exported into an Excel spreadsheet.

#### Data extraction

*First*, we will develop a data-charting form to guide extraction of variables based on the Montreal model [14], which is an adapted version of the framework for patient and family engagement in health [13]. The data-charting form will include the PICOS elements and “PROGRESS” (place of residence, race/ethnicity/culture/language, occupation, gender/sex, religion, education, socioeconomic status, and social capital) factors [44]. *Second*, two reviewers will perform a calibration exercise to guide selection of evidence sources to ensure the form captures all relevant data. Then, they will independently extract data from some included studies using the data-charting form and meet the steering committee members to determine whether the approach to data extraction is consistent with the research question and purpose. *Third*, we will separately extract strategies used for involving (1) patients and/or the public (alone) and (2) multiple stakeholders including patients and/or the public. We will also separately extract strategies used for women (e.g., for scaling up a prenatal screening program) from those used for men (e.g., for scaling up a prostate cancer program), and other sex- or gender-specific strategies. *Finally*, we will resolve any disagreements through consensus between the two reviewers and, if needed, with a third party. We will contact authors of included studies to provide missing or additional data if necessary.

#### Risk of bias

We will not perform an appraisal for risk of bias. Due to the nature of our research question, assessing risk of bias is not mandatory, according to the Joanna Briggs Institute Reviewers’ Manual [27, 45].

#### Data analysis

We will summarize data quantitatively (using frequencies) and qualitatively (drawing on the descriptive analytical method) to describe PPI strategies in scaling-up initiatives in HSS. We will structure the data on the included studies according to the PICOS elements and PROGRESS factors. We anticipate that the development of a quantitative meta-analysis will not be feasible due to the nature of our research question and substantial methodological heterogeneity of studies identified. If possible, we will stratify results by (1) economic status of the country (e.g., low-, middle-, or high-income country), (2) sex/gender, and (3) health context (e.g., primary care, secondary, and tertiary care). We will report our results using the Preferred Reporting Items for Systematic reviews and Meta-Analyses extension for Scoping Reviews (PRISMA-ScR) checklist [25], the SAGER guidelines [24], and the GRIPP2 reporting checklist [23].

#### Consultation with the steering committee

Our steering committee will seek consensus on a preliminary set of PPI strategies to present in an online Delphi (e-Delphi) survey. *First,* we will provide the committee with a list of the PPI strategies found in the literature. Each steering committee member will review the inventory and judge whether the PPI strategy represents the construct of PPI or not. *Second,* the project leaders will produce a comprehensive list of voted PPI strategies. *Third,* a videoconference will be conducted to reach a decision about which PPI strategies will be included in a preliminary list in the open round of the following Delphi study.

### Delphi study

#### Study design

As part of the RAND/UCLA Appropriateness Method [18, 19], we will use evidence from the knowledge synthesis to achieve a consensus opinion on the relevant PPI strategies for scaling-up initiatives in HSS.

#### Participants

Based on sampling trends and recommendations from previous Delphi studies, we aim to invite 100 participants [46] to help ensure that a minimum of 16 stakeholders complete all the rounds [47]. We will include at least four patients or citizens (men and women), two health care providers, two policy or decision makers, two trainees, two members of patient-oriented research organizations, two scaling-up researchers, and two first or last authors of scaling-up studies. We will include them according to (1) their age (at least 18 years old); (2) their ability to participate, read, and understand English or French; and (3) their knowledge or interest in the field. We plan to recruit participants through the directed snowball method. We will ensure patients and citizens understand our research objectives and participate in decisions as we prepare and oversee information provided to them.

#### Data collection

We anticipate that the following three rounds will allow an acceptable degree of agreement on research priorities, but if not, a fourth round will be undertaken. In each round, we will ask participants to complete their questionnaires within 2 weeks, and we will send two reminder emails before the deadline. In order to enable efficient and timely data collection with international participants, we will use the REDCap platform, a secure web application on server infrastructure physically located at the University of British Columbia University Data Centre in Canada.

- ○ Round 1: We will collect participants’ sociodemographic information (e.g., country, sex, areas of expertise, primary occupational role). For each PPI strategy included in the preliminary list, we will ask participants to (1) rate how relevant the strategy is to engaging women, men, or other sex/gender categories; (2) rate how relevant the strategy is to each of the scaling-up steps; (3) determine how relevant the strategy is to various contexts (e.g., low-, middle-, or high-income countries; primary, secondary, or tertiary care); and (4) suggest any other PPI strategies required in scaling-up initiatives in HSS. Responses will be collated and discussed by our steering committee, and a list of strategies will be drawn up for scoring in the following round.
- ○ Round 2: Participants will be asked to individually score each PPI strategy for its overall relevance to the largest range of sex/gender categories, scaling up steps and contexts. In addition, they will be invited to provide a brief justification or cite a relevant study to support their scores. Finally, there will be an opportunity to add any further PPI strategy that respondents feel should be considered. We will collate results and calculate the median score and percentage of agreement.
- ○ Round 3: We will send a checklist to participants who completed the second round in which the median results from the third round are listed alongside the participant’s own score. Participants will be invited to reconsider the relevance of the PPI strategies and confirm or revise their score in the light of the participants’ opinions.

#### Data analysis

We will use a 10-point Likert scale ranging from 0 = “not relevant” to 9 = “highly relevant.” We will categorize participants’ responses as low (0–3), moderate (4–6), and high (7–9) [48]. For each PPI strategy, consensus will be defined as 80% agreement for the priority score of 7–9. Results will be collated using Excel to calculate the median score and percentage of agreement.

### Build collaboration network

We will use a snowballing recruitment method to develop an international network of stakeholders who share an interest in the field of scaling up in HSS. The snowball method is modeled after “contact tracing” in health, in which one individual names all other individuals who were associated with a specific event [49]. We established the RePOS network in March 2020 and included over 20 members (e.g., patients, citizens, health care providers, policy makers, researchers, and trainees) from low-, middle-, and high-income countries. *First*, with our steering committee members, we will define eligibility criteria and draft an indicative electronic membership form. *Second*, we will email steering committee members, our collaborators, and all Delphi study participants to ask them to become members of the RePOS network by signing the electronic form. Also, we will ask them to suggest other stakeholders eligible for inclusion in the network. *Third*, we will use internet-based forums, conference calls, monthly e-bulletins, and ongoing exchange to increase the network’s reach to other experts in the field. We will continue to add stakeholders until we have no new eligible names. *Finally*, the creation of this group will allow us to experience all the difficulties and complexities of involving PPI and multiple stakeholders in a scaling-up project.

## Discussion

In this project, we will identify relevant strategies for meaningfully and equitably involving patients and the public in the science and practice of scaling up in HSS. Overall, outcomes will be:

1. An inventory of existing strategies related to the involvement of patients and the public in scaling-up initiatives in HSS. This inventory will serve as a guide for those planning scaling up to map their specific PPI needs;
2. A consensus on most relevant strategies for PPI in scaling-up initiatives in HSS. Analysis of common elements of relevant PPI strategies will provide a generic framework for future endeavors;
3. An international network of stakeholders working in patient-oriented research on scaling up. Overall, this collaboration will utilize and disseminate our research results (most effective PPI strategies for scaling up evidence-based innovations) to ensure that more scaling-up initiatives worldwide reflect the needs and priorities of those they are intended to benefit.

Our project will result in a better understanding of PPI in scaling-up initiatives in HSS, a knowledge gap identified in previous work [6, 50]. Our project will also contribute to knowledge about effective PPI in other complex research projects. Our findings have the potential to increase the impact of evidence-based innovations worldwide (including in lower- and middle-income countries), ensure they benefit the people they are intended for, reduce waste, and ultimately contribute to reducing health inequities and improving the health of populations.

We acknowledge that this project includes some operational challenges, such as restricted face-to-face interactions and activities because of the coronavirus disease 2019 (COVID-19) pandemic. However, we will use teleconference software to continue working as a team and involve patients, citizens, and participants who are comfortable with using these technologies. Another potential challenge common to Delphi studies is participant dropout, but we will maintain participant enthusiasm and engagement by expressing our appreciation for their time in every email correspondence, maintaining connections in person by telephone, and following-up with non-respondents [51, 52].

Finally, we will disseminate our results to knowledge users through publications in peer-reviewed journals, presentations at relevant conferences, and organization of workshops. We will also share our reports using free public repositories such as Open Science Framework and ResearchGate. The dissemination of this project will start with the publication of the protocol. Any amendments made to this protocol will be reported in the final manuscript.

## Supplementary Information

**Additional file 1.** PRISMA-P 2015 Checklist.

**Additional file 2.** Search strategy.

## Data Availability

Please send all requests for study data or materials to Dr. Ali Ben Charif (ali.bencharif@gmail.com) and Dr. France Légaré (france.legare@mfa.ulaval.ca).

## Abbreviations

AHRQ: Healthcare Research and Quality
CADTH: Canadian Agency for Drugs and Technologies in Health
CIHR: Canadian Institutes of Health Research
EBI: Evidence-based innovation
GRIPP2: Guidance for Reporting Involvement of Patients and the Public
HSS: Health and social services
iKT: Integrated knowledge translation
JBI: Joanna Briggs Institute
KT: Knowledge translation
OSF: Open Science Framework
PICOS: Participants, intervention, comparator, outcome, and setting
PPI: Patient and public involvement
PPEET: Public and Patient Engagement Evaluation Tool
PRISMA: Preferred Reporting Items for Systematic reviews and Meta-Analyses
PRISMA-P: Preferred Reporting Items for Systematic reviews and Meta-Analyses Protocol
PRISMA-ScR: Preferred Reporting Items for Systematic reviews and Meta-Analyses extension for Scoping Reviews
PRESS: Peer Review of Electronic Search Strategies
PROGRESS: Place of residence, race/ethnicity/culture/language, occupation, gender/sex, religion, education, socioeconomic status, and social capital
RePOS: Research on patient-oriented scaling-up
SAGER: Sex and Gender Equity in Research
